# Supplementary material for: Structural insight into hierarchical DNMT3A autoinhibition and its dysregulation in disease
Source: Nat Commun. 2026 Feb 18;17:2901. doi: 10.1038/s41467-026-69563-1 (PMC13031684; doi:10.1038/s41467-026-69563-1)
Supplement: Supplementary file 1 — Supplementary information [file 41467_2026_69563_MOESM1_ESM.pdf]

Supplementary information for

Structural insight into hierarchical DNMT3A autoinhibition and its dysregulation in disease

Jiuwei Lu[1], Emily Vig[2,#], Jianbin Chen[1,2,#], Kristjan H. Gretarsson[3,#], Nelli Khudaverdyan[1,2], Zengyu Shao[1,2], Chao Lu[3], Chia-en A. Chang[2,4], Jikui Song[1,2,*]

[1]Department of Biochemistry, University of California, Riverside, CA 92521, USA

[2]Biochemistry and Molecular Biology Graduate Program, University of California, Riverside, CA 92521, USA

[3]Department of Genetics and Development and Herbert Irving Comprehensive Cancer Center, Columbia University Irving Medical Center, New York, NY 10032, USA.

[4]Department of Chemistry, University of California, Riverside, CA 92521, USA

[#]These authors contributed to the project equally

*Correspondence: jikui.song@ucr.edu

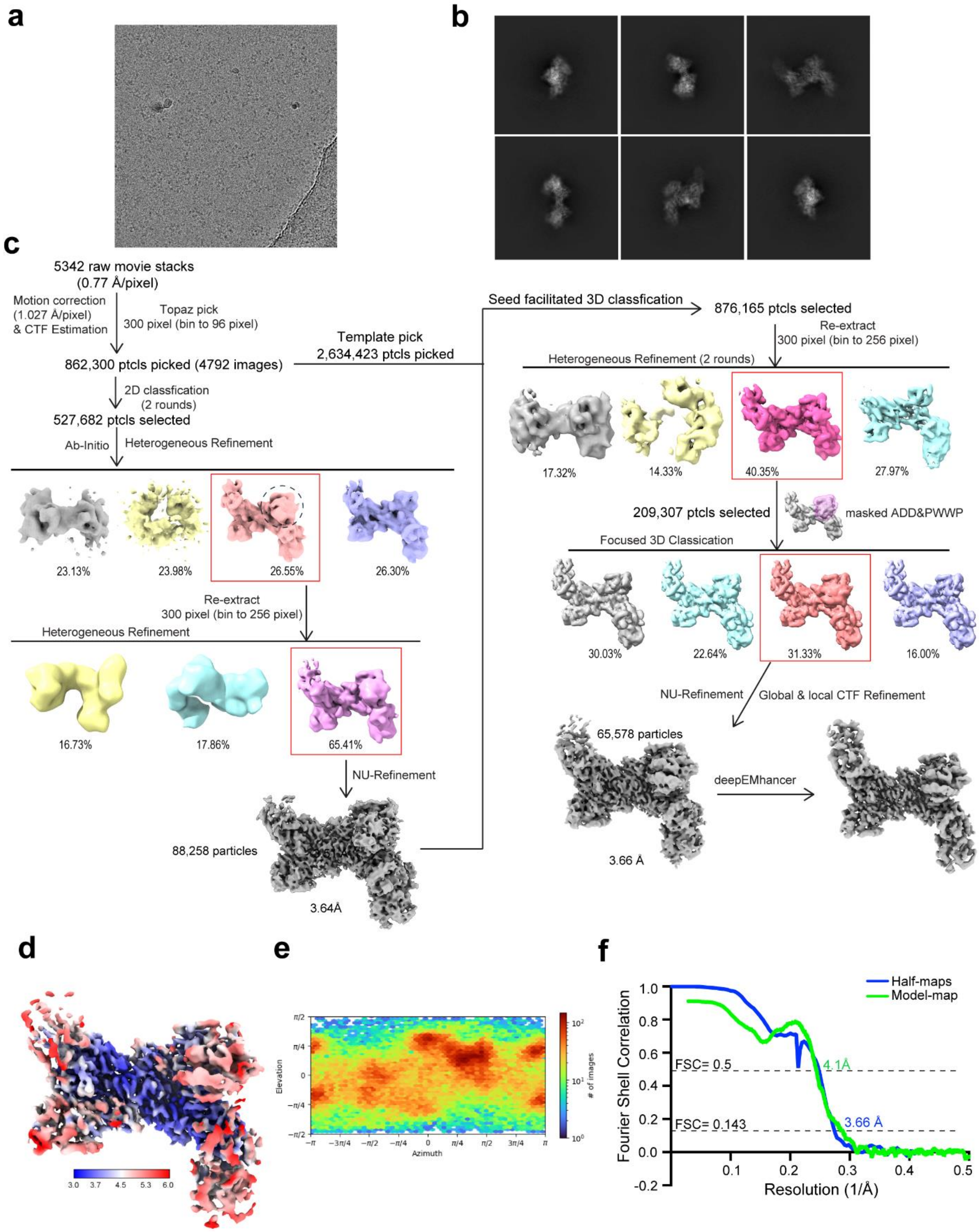

Supplementary Figure 1. Cryo-EM structure of the DNMT3A2-DNMT3L complex. a A representative micrograph of the DNMT3A2-DNMT3L complex by Cryo-EM. **b** Representative 2D classes of the DNMT3A2-DNMT3L complex from cryoSPARC. **c**

Workflow for data processing**.** The particle groups used for subsequent data processing were indicated by red squares. For instance, after initial heterogeneous refinement, the particles associated with the density with the feature of the PWWP domain (circled) were selected for further classification. **d-f** Local resolution (d), Angular distribution of final particles used for construction (e), and FSC curve for half maps and model-map for the structure of the DNMT3A2-DNMT3L complex (f).

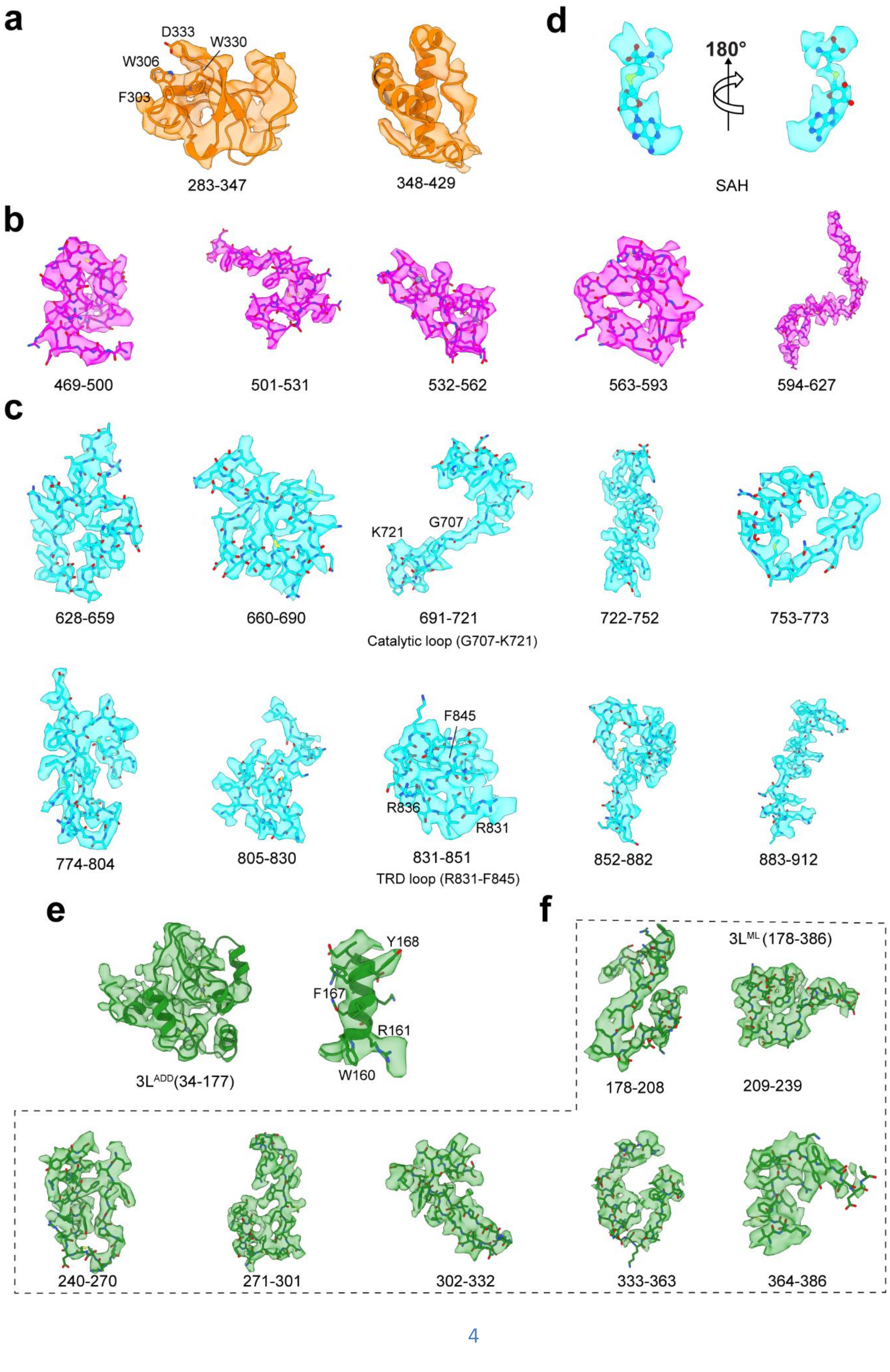
a
D333
W330
W306
F303
283-347
348-429
d
180°
SAH
b
469-500
501-531
532-562
563-593
594-627
c
628-659
660-690
K721
G707
691-721
Catalytic loop (G707-K721)
722-752
753-773
774-804
805-830
F845
R836
R831
831-851
TRD loop (R831-F845)
852-882
883-912
e
Y168
F167
R161
W160
3L^ADD(34-177)
f
3L^ML (178-386)
178-208
209-239
240-270
271-301
302-332
333-363
364-386

Supplementary Figure 2. Cryo-EM density maps of the DNMT3A2-DNMT3L complex. **a-d** Density maps for representative regions of the PWWP domain (a), ADD domain (b), MTase domain (c), and bound SAH (two opposite views) (d) of DNMT3A. **e,f** Density maps for representative structural elements of the ADD domain (e) and ML domain (f) of DNMT3L.

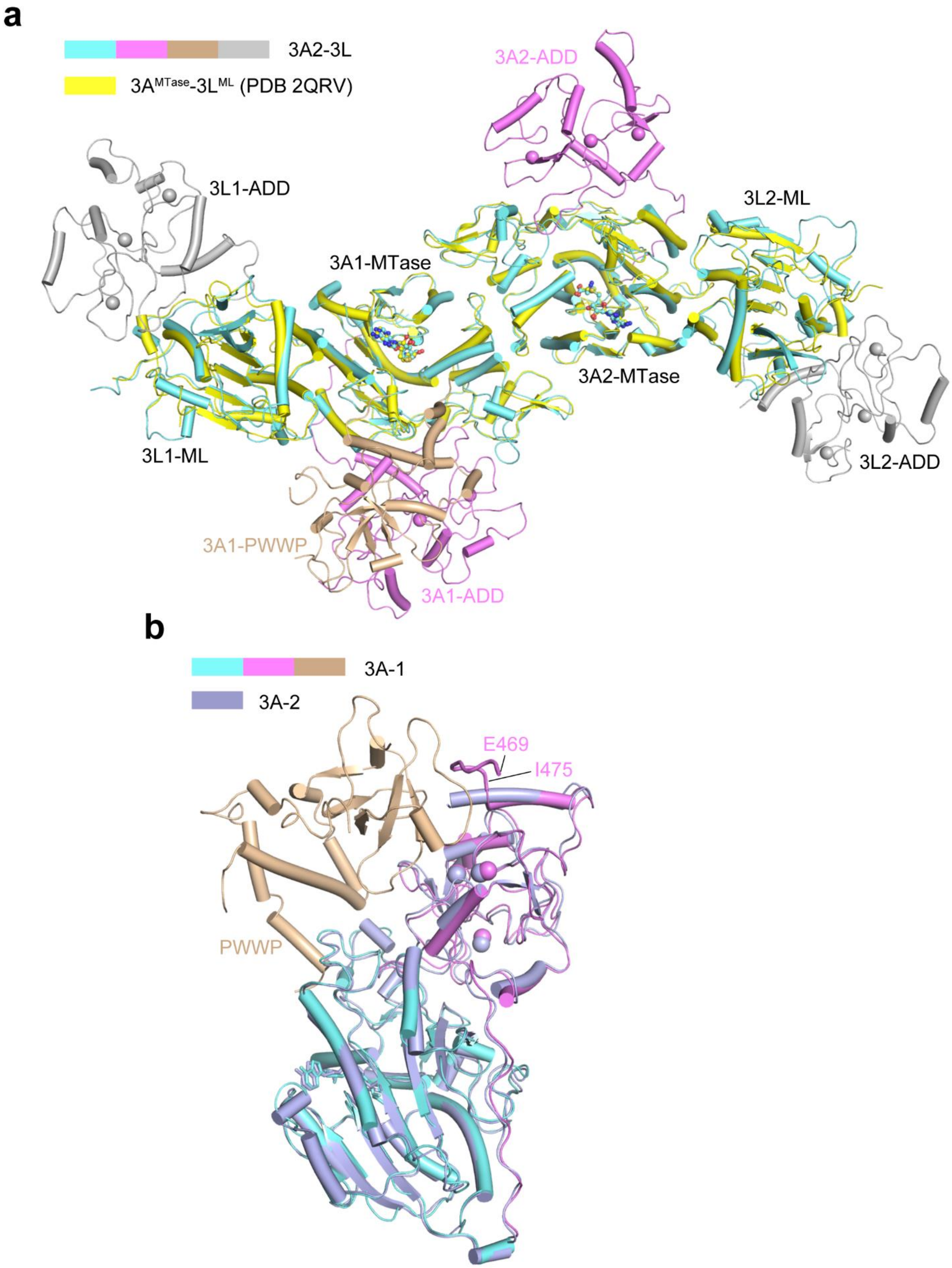
a
3A2-3L
3A^{MTase}-3L^{ML} (PDB 2QRV)
3A2-ADD
3L1-ADD
3L2-ML
3A1-MTase
3A2-MTase
3L1-ML
3L2-ADD
3A1-PWWP
3A1-ADD
b
3A-1
3A-2
E469
I475
PWWP

Supplementary Figure 3. Structural overview of the DNMT3A2-DNMT3L complex. a Structural overlay of the DNMT3A2-DNMT3L complex with the previously reported DNMT3A^{MTase}-DNMT3L^{ML} (PDB 2QRV), with the domains of individual subunits labeled. **b** Structural overlay of the 3A-1 and 3A-2 subunits in the DNMT3A2-DNMT3L complex.

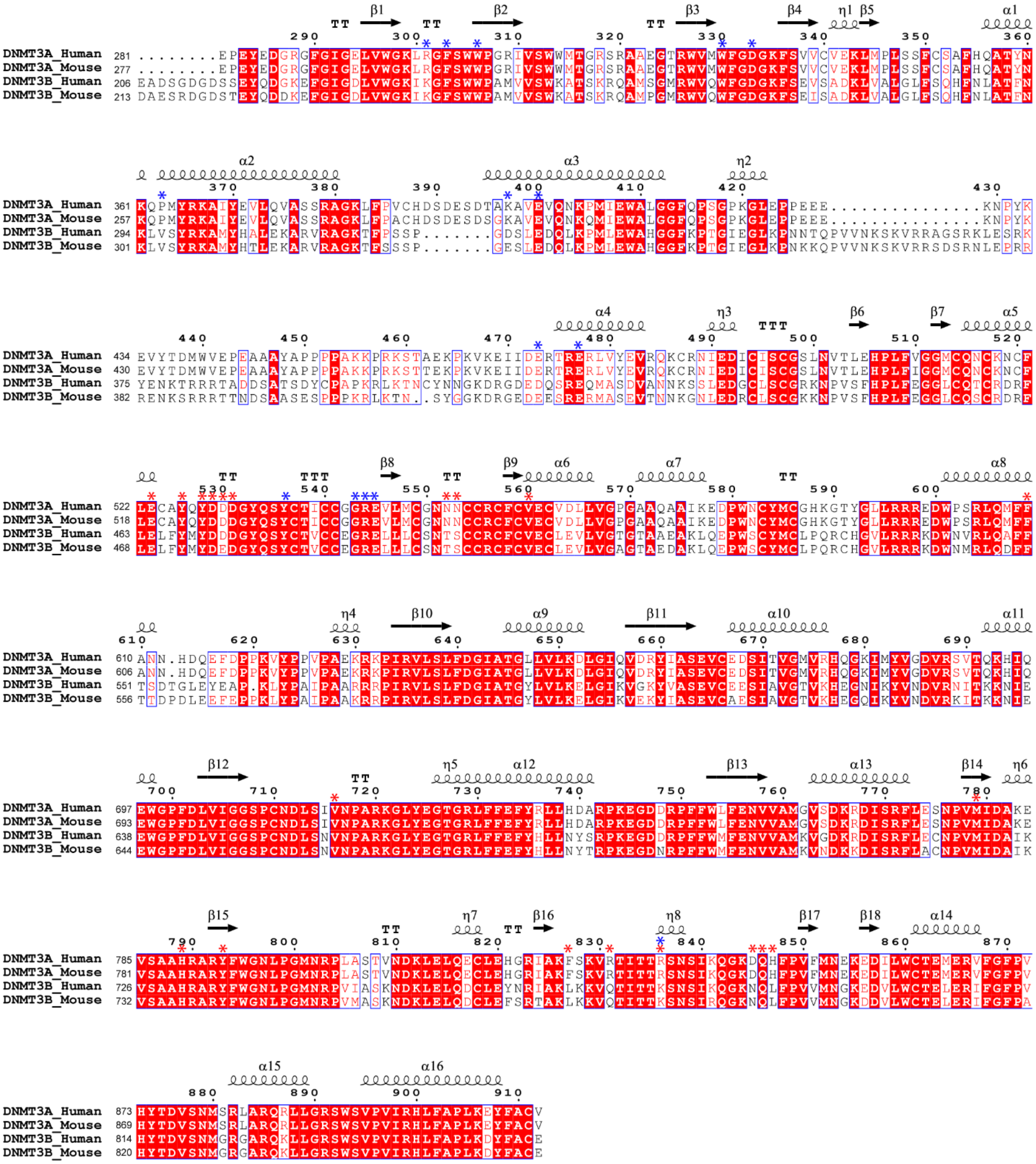

Supplementary Figure 4. Structure-based sequence alignment of human and mouse DNMT3A and DNMT3B. Identical or similar residues are boxed and colored in red. Completely conserved residues are shaded in red. Identical residues are colored white in red background and similar residues are colored in red. The residues involved in the PWWP-mediated interaction with the ADD and MTase domains and the binary ADD-

MTase interaction are marked by blue and red asterisks, respectively, on top. The secondary structures corresponding to DNMT3A are also indicated on top.

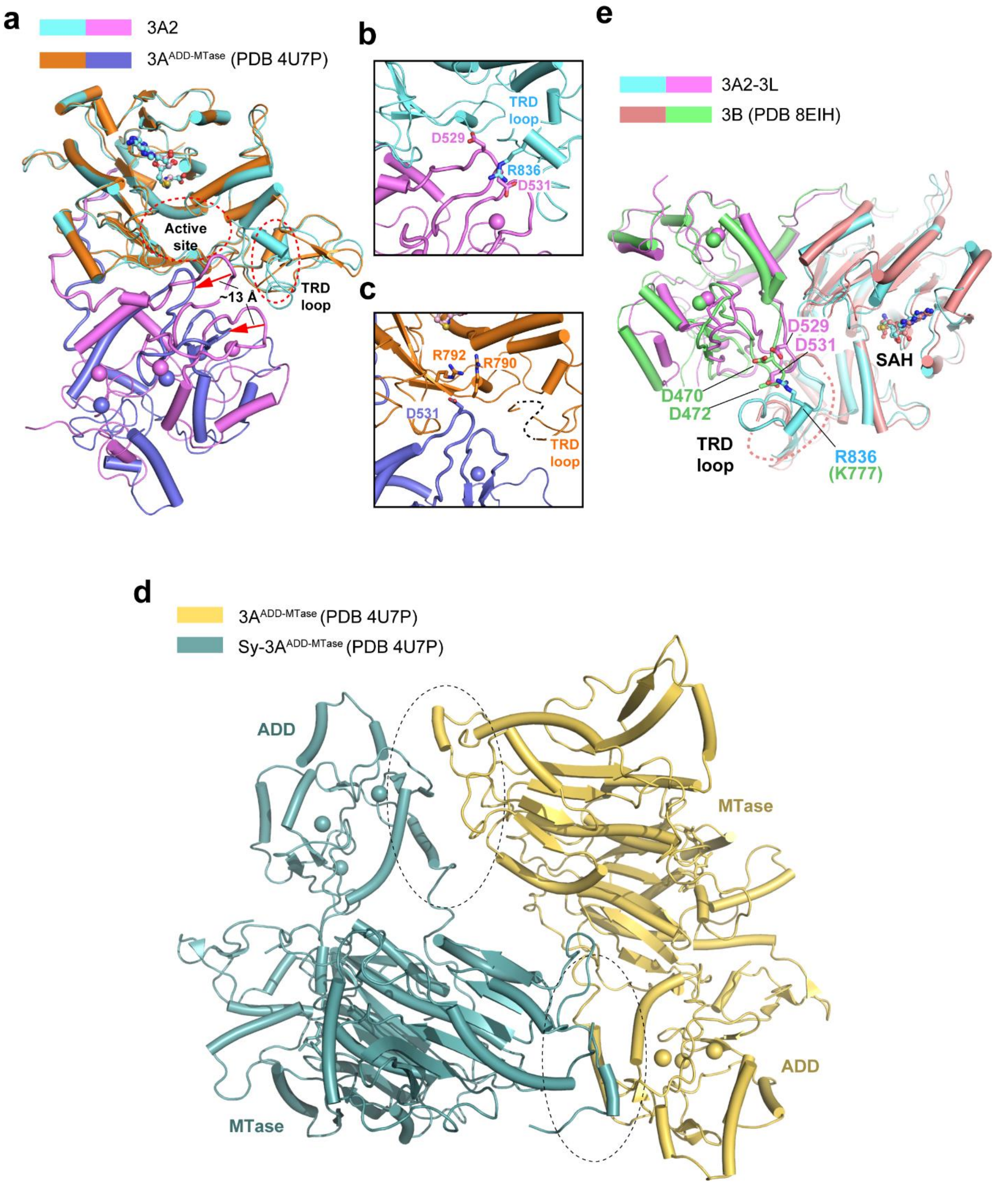

Supplementary Figure 5. Structural comparison between the cryo-EM structure of DNMT3A2-DNMT3L and the crystal structure of DNMT3A$^{ADD\text{-}MTase}$-DNMT3L^{ML}. a Structural overlay of the DNMT3A subunit in the DNMT3A2-DNMT3L complex and the

DNMT3A$^{ADD-MTase}$-DNMT3L^{ML} complex (PDB 4U7P), with a ~13-Å shift of the ADD positioning indicated by red arrows. **b,c** Close-up view of the ADD-MTase interaction in the DNMT3A2-DNMT3L complex (b) and the DNMT3A$^{ADD-MTase}$-DNMT3L^{ML} complex (PDB 4U7P) (c). Note that the ADD residue D531 interacts with TRD-loop residue R836 in (b) but residues R790 and R792 in (c). **d** Crystal packing analysis of the DNMT3A$^{ADD-MTase}$-DNMT3L^{ML} complex (3A$^{ADD-MTase}$) (PDB 4U7P) and the symmetry-related complex (Sy-3A$^{ADD-MTase}$), revealing that the ADD domain and its downstream linker (marked by dotted circle) are involved in crystal packing. **e** Structural overlay of between the ADD-MTase region of the 3A-1 subunit and the corresponding region in DNMT3B homotetramer (PDB 8EIH). Note that the ADD domains are positioned similarly between the two proteins. The TRD loop of DNMT3B is shown as dashed line due to disorder. The interacting residues, DNMT3A D529, D531 and R836, and the corresponding sites in DNMT3B shown in stick representation. DNMT3B K777, corresponding to DNMT3A R836, is not shown due to structural disorder.

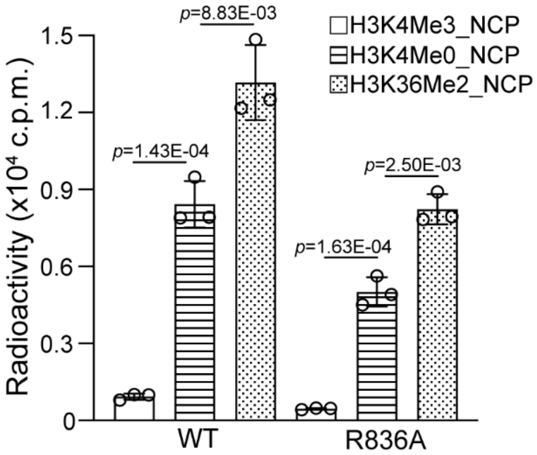

Supplementary Figure 6. *In vitro* DNA methylation assay of DNMT3A2-DNMT3L, WT or R836A mutant, on the nucleosome substrates with various histone modifications. The statistical analysis used two-tailed Student's t test. Data are mean $\pm$ s.d. (n = 3 biological repeats). Source data are provided as a Source Data file.

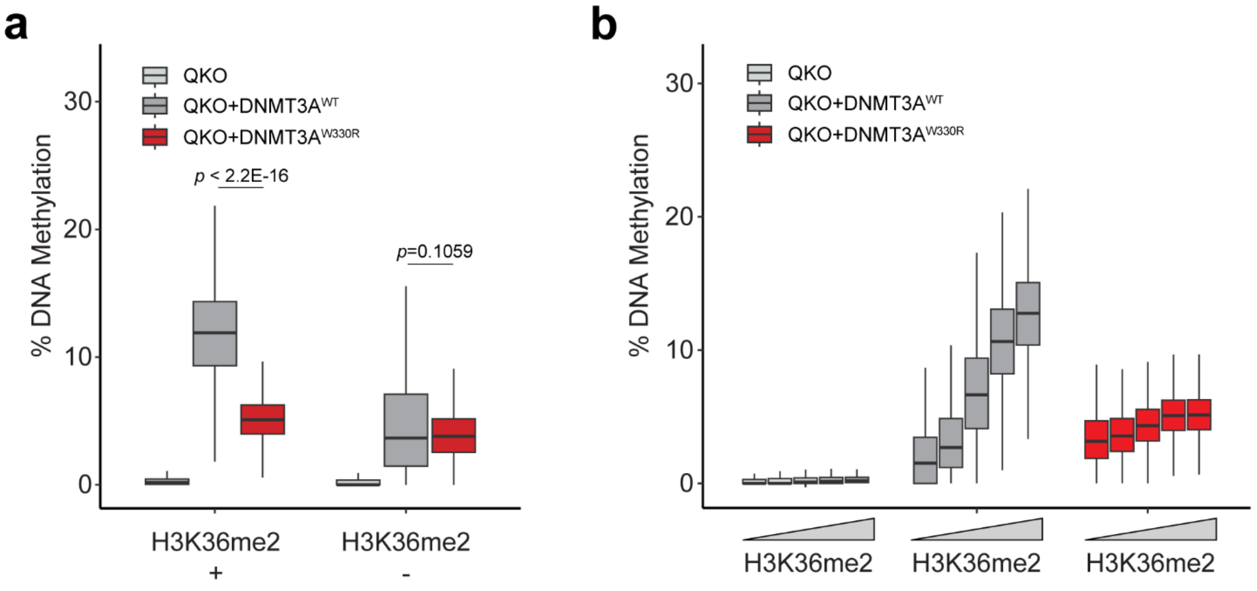

Supplementary Figure 7. Genomic DNA methylation analysis of QKO mESCs with WT and W330R DNMT3A1. a Box plots showing the levels of DNA methylation in CGI-excluded, H3K36me2-positive (left) or negative (right) regions in QKO reconstituted with WT or W330R DNMT3A1. Box-and whisker plots depict 25-75% in the box, whiskers are 1.5 times the interquartile range (IQR), and median is indicated. Wilcoxon rank-sum two-sided test is used for statistical analysis (n = 2 biological replicates). **b** Percent of DNA methylation in WT or W330R DNMT3A1 QKO mESCs grouped by H3K36me2 CUT&RUN enrichments. Box-and whisker plots depict 25-75% in the box, whiskers are 1.5 times the interquartile range (IQR), and median is indicated (n = 2 biological replicates).

Supplementary Table 1. Cryo-EM data collection, structure refinement and validation statistics

States	DNMT3A/3L (EMD-71814, PDB 9PRW)
**Data collection and processing**	
Microscope	Titan Krios
Camera	TFS Falcon IV
Magnification	130,000
Voltage (kV)	300
Defocus range (μm)	-0.8 ~ -2.5
Exposure time (s)	7
Dose rate(e^{-}/*px*/s)	7 e^{-}/px/s
Number of frames	29
Pixel size (Å)	0.7675
Micrographs (no.)	5342
Initial particles (no.)	2,634,423
Symmetry imposed	*C1*
Final particles (no.)	65,578
Map resolution (Å)	3.66
FSC threshold	0.143
**Refinement**	
Initial model used	4U7P,3LLR, 2PV0
Model resolution (Å)	4.1
FSC threshold	0.5
Map sharpening *B* factor (Å^2)	deepEMhanceer (visualization)<br>uniform B-factor of -116.28Å^2 (refinement)
Model composition	
Non-hydrogen atoms	13,546
Protein residues	1703
Ion (zinc)	12
SAH	2
B factors (Å^2)	
Protein	123.4
Ion (zinc)	205.1
SAH	47.8
R.m.s. deviations	
Bond lengths (Å)	0.003
Bond angles (°)	0.661
**Validation**	
MolProbity score	1.9
Clashscore	9.98
Poor rotamers (%)	0.14
Ramachandran plot	
Favored (%)	94.38
Allowed (%)	5.62
Disallowed (%)	0

Supplementary Table 2. List of primers used in this study

Name	Sequence
3A_sy_281F_VP13	GTACATCCAAGGATCCGAACCGGAATATGAAGATGGT
3A_sy_912R_VP13	GTCCTACAGGCGCGCCTTAAACGCAGGCAAAATATTCTTT
3L_1F_Vp13	GTACATCCAAGGATCCATGGCGGCCATCCCAGCCCTGGA
3L_AscII_386R	GTCCTACAGGCGCGCCTTAGGGTTGGTGTTGAGGAGACT
3A_281F_pRSF_sy	ACAGATTGGTGGATCCGAACCGGAATATGAAGATGGT
3A_427R_pRSF_sy	CTTTACCAGACTCGAGTTATTCTTCAGGCGGTTCCAGACC
3A_476F_pRSF_sy	ACAGATTGGTGGATCCCGTGAACGTCTGGTTTATGAA
3A_614R_pRSF_sy	CTTTACCAGACTCGAGTTAATCGTGGTTATTTGCAAAAAA
hDNMT3A_912Not1	TAAGCATTATGCGGCCGCTTAAACGCAGGCAAAATATTC
3A_W330A_SyF	TGGGTTATGGCGTTTGGTGATGGTAAATTTAGC
3A_W330A_SyR	CATCACCAAACGCCATAACCCAACGGGTGCCTT
3A_W330R_SyF	TGGGTTATGCGTTTTGGTGATGGTAAATTTAGC
3A_W330R_SyR	CCATCACCAAAACGCATAACCCAACGGGTGCCT
3A_D333N_syF	TGGTTTGGTAACGGTAAATTTAGCGTTGTGTGC
3A_D333N_syR	TAAATTTACCGTTACCAAACCACATAACCCAAC
3A_R544A_syF	TGTGGTGGTGCTGAAGTTCTGATGTGTGGTAAT
3A_R544A_syR	TCAGAACTTCAGCACCACCACAACAAATGGTGC
3A_E545G_syF	GGTGGTCGTGGCGTTCTGATGTGTGGTAATAAC
3A_E545G_syR	ACATCAGAACGCCACGACCACCACAACAAATGG
3A_R836A_syF	ATTACCACCGCGAGCAATAGCATTAAACAGGGT
3A_R836A_syR	TGCTATTGCTCGCGGTGGTAATGGTACGAACTT
3A_D529D531AA_syF	TATCAGTATGCGGATGCCGGTTATCAGAGCTAT
3A_D529D531AA_syR	TCTGATAACCGGCATCCGCATACTGATAGGCGC
DNMT3L_pRSF_178F	AAGGAGATATACATATGATGTTCGAAACCGTGCCTGTGT
DNMT3L_pRSF_386R	CTTTACCAGACTCGAGTTATAAAGAGGAAGTGAGTTCTGTT

Supplementary Table 3. Setup of molecular dynamics simulation

System	Water box (12 Å)	Water molecules	Na+ Ions added	Total Number of Atoms
DNMT3A $^{PWWP-ADD-MTase}$	12	91759	25	302350
DNMT3A $^{ADD-MTase}$	12	92490	28	302153
DNMT3A $^{ADD-MTase}$-H3	12	92430	22	302243
