# Supplementary material for: Structural insight into hierarchical DNMT3A autoinhibition and its dysregulation in disease
Source: Nat Commun. 2026 Feb 18;17:2901. doi: 10.1038/s41467-026-69563-1 (PMC13031684; doi:10.1038/s41467-026-69563-1)
Supplement: Supplementary file 2 — Description of additional supplementary file [file 41467_2026_69563_MOESM2_ESM.pdf]

Description of Additional Supplementary Files

Supplementary Data 1

Description: Initial and final configurations of the DNMT3A$^{PWWP\text{-}ADD\text{-}MTase}$, DNMT3A$^{ADD\text{-}MTase}$ and DNMT3A$^{ADD\text{-}MTase}$-H3 complexes in MD simulation.
