# Supplementary material for: Structural insight into hierarchical DNMT3A autoinhibition and its dysregulation in disease
Source: Nat Commun. 2026 Feb 18;17:2901. doi: 10.1038/s41467-026-69563-1 (PMC13031684; doi:10.1038/s41467-026-69563-1)
Supplement: Supplementary file 4 — Reporting Summary [file 41467_2026_69563_MOESM4_ESM.pdf]

nature portfolio

Corresponding author(s): Jikui Song

Last updated by author(s): Jan 23, 2026

Reporting Summary

Nature Portfolio wishes to improve the reproducibility of the work that we publish. This form provides structure for consistency and transparency in reporting. For further information on Nature Portfolio policies, see our Editorial Policies and the Editorial Policy Checklist.

Statistics

For all statistical analyses, confirm that the following items are present in the figure legend, table legend, main text, or Methods section.

n/a	Confirmed	
☐	☒	The exact sample size (*n*) for each experimental group/condition, given as a discrete number and unit of measurement
☐	☒	A statement on whether measurements were taken from distinct samples or whether the same sample was measured repeatedly
☐	☒	The statistical test(s) used AND whether they are one- or two-sided<br>*Only common tests should be described solely by name; describe more complex techniques in the Methods section.*
☒	☐	A description of all covariates tested
☒	☐	A description of any assumptions or corrections, such as tests of normality and adjustment for multiple comparisons
☐	☒	A full description of the statistical parameters including central tendency (e.g. means) or other basic estimates (e.g. regression coefficient) AND variation (e.g. standard deviation) or associated estimates of uncertainty (e.g. confidence intervals)
☐	☒	For null hypothesis testing, the test statistic (e.g. *F*, *t*, *r*) with confidence intervals, effect sizes, degrees of freedom and *P* value noted<br>*Give P values as exact values whenever suitable.*
☒	☐	For Bayesian analysis, information on the choice of priors and Markov chain Monte Carlo settings
☒	☐	For hierarchical and complex designs, identification of the appropriate level for tests and full reporting of outcomes
☒	☐	Estimates of effect sizes (e.g. Cohen's *d*, Pearson's *r*), indicating how they were calculated

Our web collection on statistics for biologists contains articles on many of the points above.

Software and code

Policy information about availability of computer code

Data collection	Cryo-EM data were collected using SerialEM from Pacific Northwest Center for Cryo-EM (PNCC). NMR spectroscopy was performed on a 700 MHz spectrometer (Bruker Avance) ;
Data analysis	For structural study, the cryoSPARC(v4.4.1), PHENIX v1.21.2_5419, Coot v0.9.8, Pymol v2.6.2, Chimera (v1.17.3) and ChimeraX (1.9) softwares were used for data processing and analysis. For NMR data processing, the TopSpin (Bruker BioSpin) and Mnova NMR (Mestrelab Research) were used. AMBER and ZAFF were used for MD simulation and visualized by VMD. For enzymatic activity comparsion and Fluorescence Polarization analysis, data were plotted and fitted using GraphPad Prism (v.10). For ITC experiment, the data were processed and fitted in Origin (MicroCal). For CUT&RUN and Reduced representation bisulfite sequencing (EM-seq) data analysis, the DeepTools (v3.5.1), SEACR (v1.3) and methylkit (v1.26) were used.

For manuscripts utilizing custom algorithms or software that are central to the research but not yet described in published literature, software must be made available to editors and reviewers. We strongly encourage code deposition in a community repository (e.g. GitHub). See the Nature Portfolio guidelines for submitting code & software for further information.

Data

Policy information about availability of data

All manuscripts must include a data availability statement. This statement should provide the following information, where applicable:

- Accession codes, unique identifiers, or web links for publicly available datasets
- A description of any restrictions on data availability
- For clinical datasets or third party data, please ensure that the statement adheres to our policy

The atomic model for the DNMT3A2-DNMT3L complex has been deposited in the Protein Data Bank under accession code 9PRW [https://www.wwpdb.org/pdb?id=pdb_00009prw]. The cryo-EM density map has been deposited in EMDB under the accession number of EMD-71814 [https://www.ebi.ac.uk/emdb/EMD-71814]. The PDB accession codes 2PVC [https://doi.org/10.2210/pdb2pvc/pdb], 2PV0 [https://doi.org/10.2210/pdb3llr/pdb] 3LLR [https://doi.org/10.2210/pdb3llr/pdb], 4QBQ [https://doi.org/10.2210/pdb4qbq/pdb], 4U7P [https://doi.org/10.2210/pdb4u7p/pdb], 5CIU [https://doi.org/10.2210/pdb5ciu/pdb], 5YX2 [https://doi.org/10.2210/pdb5YX2/pdb] and 8EIH https://doi.org/10.2210/pdb8eih/pdb] were used in this study. The Cut&Tag and DNA methylation profiling data deposited in NCBI Gene Expression Omnibus under accession number GSE247019 [https://www.ncbi.nlm.nih.gov/geo/query/acc.cgi?acc=GSE247019] were used in this study. Source Data are provided in the Source Data file.

Research involving human participants, their data, or biological material

Policy information about studies with human participants or human data. See also policy information about sex, gender (identity/presentation), and sexual orientation and race, ethnicity and racism.

Reporting on sex and gender	N/A.
Reporting on race, ethnicity, or other socially relevant groupings	N/A.
Population characteristics	N/A.
Recruitment	N/A.
Ethics oversight	N/A.

Note that full information on the approval of the study protocol must also be provided in the manuscript.

Field-specific reporting

Please select the one below that is the best fit for your research. If you are not sure, read the appropriate sections before making your selection.

☒ Life sciences ☐ Behavioural & social sciences ☐ Ecological, evolutionary & environmental sciences

For a reference copy of the document with all sections, see nature.com/documents/nr-reporting-summary-flat.pdf

Life sciences study design

All studies must disclose on these points even when the disclosure is negative.

Sample size	The data size for Cryo-EM was determined by the availability of the microscope time and the particle density on the grids. Sufficient cryo-EM data were collected to achieve the reported resolution the map, which is sufficient for the model building. The Biochemical and enzymatic assays were completed using wild type or mutants of DNMT3A-DNMT3L. The sample size is sufficient to delineate the mutational effects of DNMT3A.
Data exclusions	Poor-quality or damaged particles were removed during Cryo-EM data processing to achieve high resolution maps which according to pre-established standard data processing procedures.
Replication	For in vitro DNA methylation and FP assays, three independent measurements were performed for each sample and stated in figure legends. For ITC assays, two independent measurements were performed with consistent binding affinity. For MD simulation, each system was performed triplicate with consistent results. NMR spectroscopy and Cryo-EM data processing was not repeated but are expected to be reproducible following the same procedure.
Randomization	This is not relevant to our study as no grouping was needed.
Blinding	Blinding is not applicable in this study.

Reporting for specific materials, systems and methods

We require information from authors about some types of materials, experimental systems and methods used in many studies. Here, indicate whether each material, system or method listed is relevant to your study. If you are not sure if a list item applies to your research, read the appropriate section before selecting a response.

Materials & experimental systems

n/a	Involved in the study
☒	☐ Antibodies
☒	☐ Eukaryotic cell lines
☒	☐ Palaeontology and archaeology
☒	☐ Animals and other organisms
☒	☐ Clinical data
☒	☐ Dual use research of concern
☒	☐ Plants

Methods

n/a	Involved in the study
☒	☐ ChIP-seq
☒	☐ Flow cytometry
☒	☐ MRI-based neuroimaging

Plants

Seed stocks	N/A.
Novel plant genotypes	N/A.
Authentication	N/A.
